# Supplementary figures and images for: Demonstration of laser biospeckle method for speedy in vivo evaluation of plant-sound interactions with arugula
Source: PLoS One. 2021 Oct 28;16(10):e0258973. doi: 10.1371/journal.pone.0258973 (PMC8553064; doi:10.1371/journal.pone.0258973)

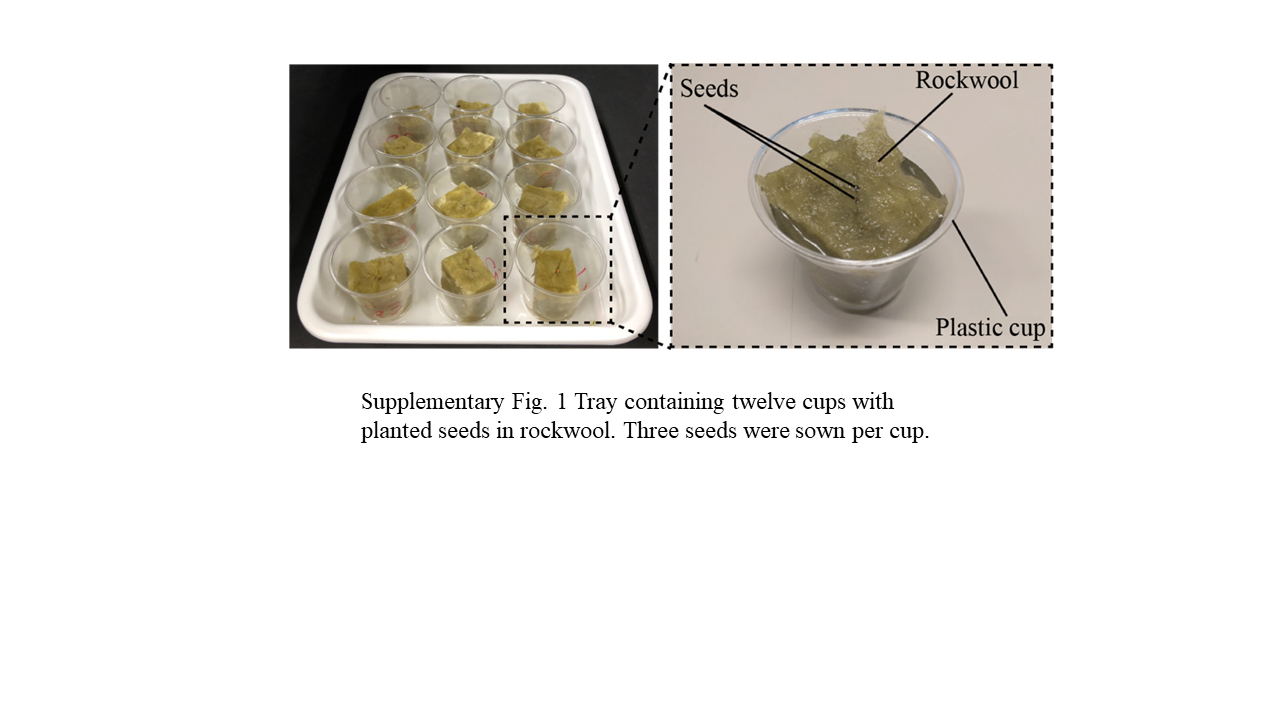

Supplement: S1 Fig — Three seeds were sown per cup. (TIF) [file pone.0258973.s001.tif]

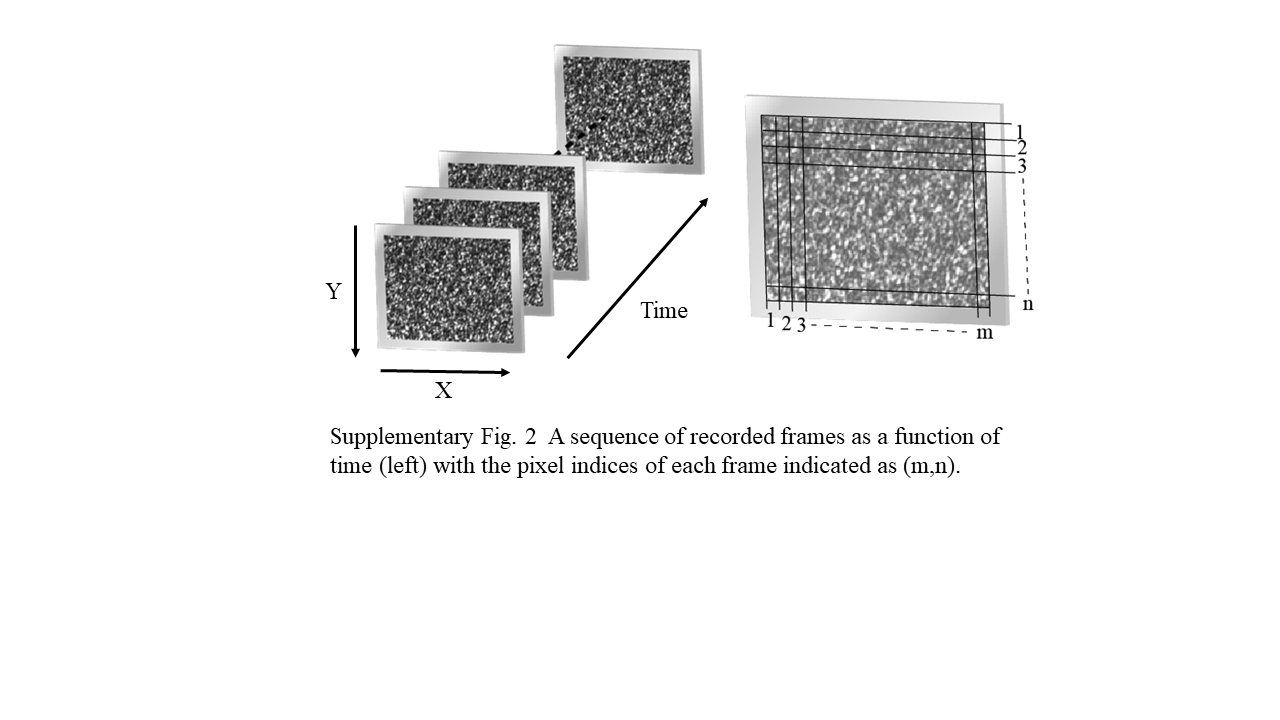

Supplement: S2 Fig — A sequence of recorded frames as a function of time (left) with the pixel indices of each frame indicated as (m,n). (TIF) [file pone.0258973.s002.tif]

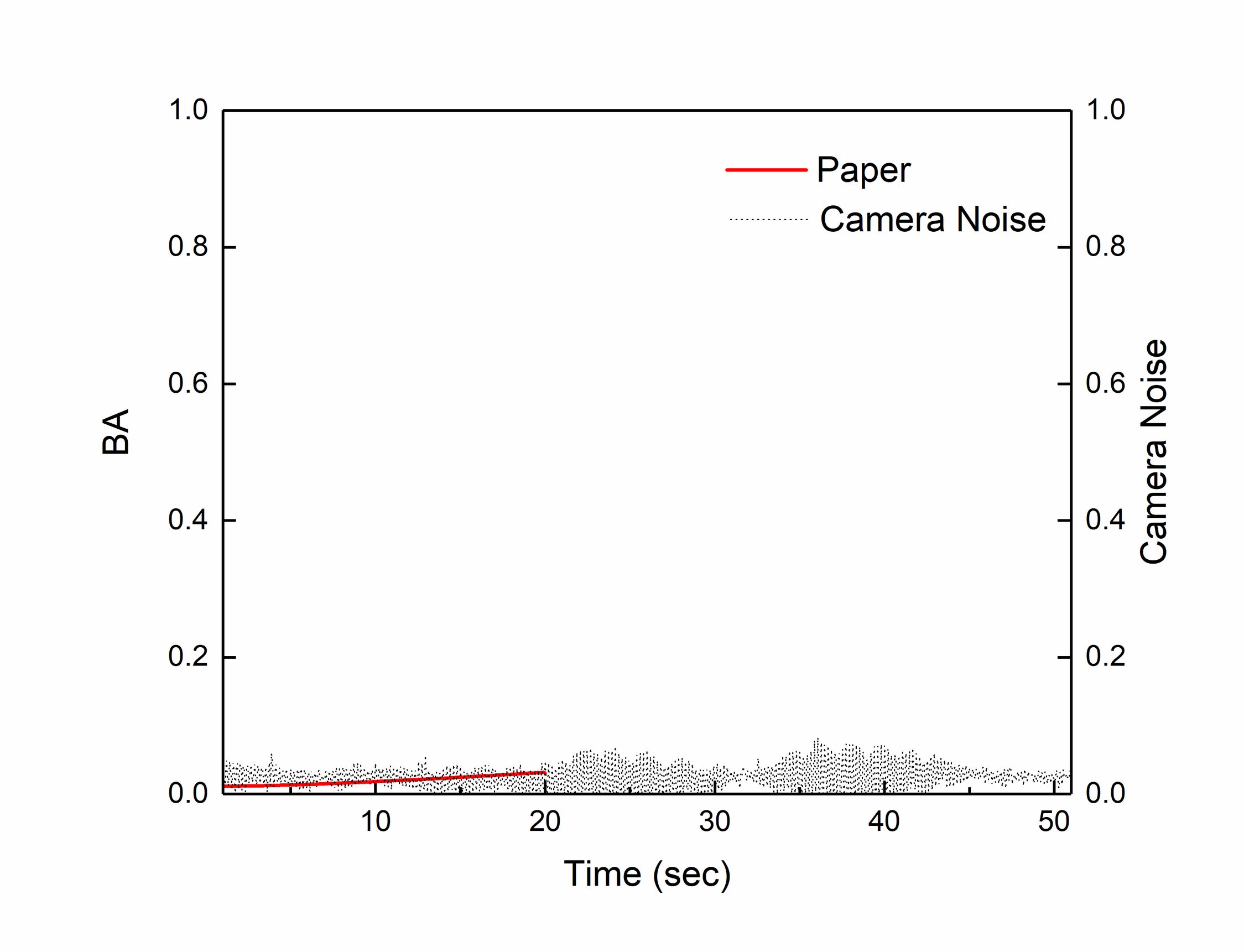

Supplement: S3 Fig — (TIF) [file pone.0258973.s003.tif]
