# Supplementary material for: Demonstration of laser biospeckle method for speedy in vivo evaluation of plant-sound interactions with arugula
Source: PLoS One. 2021 Oct 28;16(10):e0258973. doi: 10.1371/journal.pone.0258973 (PMC8553064; doi:10.1371/journal.pone.0258973)
Supplement: S1 Table — (DOCX) [file pone.0258973.s004.docx]

S1 Table

Supplementary Table of p-values of the results of t-test done under different frequencies with that under control of no sound for ages of two different leaves.

| **t-test done with control for** | **14days leaf** | **30 days leaf** |
| --- | --- | --- |
| 100Hz | 7.6x10^-8^ | 3.8x10^-2^ |
| 1kHz | 1.7x10^-4^ | 6.410^-3^ |
| 10kHz | 2.9x10^-12^ | 7.7x10^-22^ |
