# Supplementary material for: Demonstration of laser biospeckle method for speedy in vivo evaluation of plant-sound interactions with arugula
Source: PLoS One. 2021 Oct 28;16(10):e0258973. doi: 10.1371/journal.pone.0258973 (PMC8553064; doi:10.1371/journal.pone.0258973)
Supplement: Attachment — Submitted filename: Umaetal_ResponseToReviewers.docx [file pone.0258973.s005.docx]

Reviewers’ comments

Answers are indicated in black at a larger font size and the modifications in the manuscript are given in blue.

Reviewers' comments:

Reviewer's Responses to Questions

Comments to the Author

1. Is the manuscript technically sound, and do the data support the conclusions?

The manuscript must describe a technically sound piece of scientific research with data that supports the conclusions. Experiments must have been conducted rigorously, with appropriate controls, replication, and sample sizes. The conclusions must be drawn appropriately based on the data presented.

Reviewer #1: Yes

Reviewer #2: Partly

We sincerely thank the reviewers for devoting their valuable time in critically reading the manuscript and making appropriate comments and criticisms. We would like to address in the following section the issues raised by the reviewers and make changes in the revised version of the manuscript to accommodate the reviewers’ comments

2. Has the statistical analysis been performed appropriately and rigorously?

Reviewer #1: No

Reviewer #2: No

In the revised manuscript, we have included a statistical analysis to validate our results by conducting valid statistical tests. We have conducted a t-test of the data obtained under different frequencies against control or no sound and we could get significant levels *p* less than almost for all conditions below 95%.

Table 1

Supplementary Table of *p*-values of the results of *t-test* done under different frequencies with that under control or no sound for ages of two different leaves.

| **t-test done with control** | **14dap** | **30 dap** |
| --- | --- | --- |
| 100Hz | 7.6x10^-8^ | 3.8x10^-2^ |
| 1kHz | 1.7x10^-4^ | 6.410^-3^ |
| 10kHz | 2.9x10^-12^ | 7.7x10^-22^ |

3. Have the authors made all data underlying the findings in their manuscript fully available?

The PLOS Data policy requires authors to make all data underlying the findings described in their manuscript fully available without restriction, with rare exception (please refer to the Data Availability Statement in the manuscript PDF file). The data should be provided as part of the manuscript or its supporting information, or deposited to a public repository. For example, in addition to summary statistics, the data points behind means, medians and variance measures should be available. If there are restrictions on publicly sharing data—e.g. participant privacy or use of data from a third party—those must be specified.

Reviewer #1: Yes

Reviewer #2: Yes

We do not have any objection in sharing our data and we have already posted a typical data obtained in the experiment that had been analyzed to obtain correlation and thus biospeckle activity of plants under exposure to sound.

4. Is the manuscript presented in an intelligible fashion and written in standard English?

PLOS ONE does not copyedit accepted manuscripts, so the language in submitted articles must be clear, correct, and unambiguous. Any typographical or grammatical errors should be corrected at revision, so please note any specific errors here.

Reviewer #1: Yes

Reviewer #2: No

As correctly pointed out by the reviewer, we realized some grammatical mistakes. We assure that the manuscript after revision will be carefully read to be devoid of errors.

5. Review Comments to the Author

Please use the space provided to explain your answers to the questions above. You may also include additional comments for the author, including concerns about dual publication, research ethics, or publication ethics. (Please upload your review as an attachment if it exceeds 20,000 characters)

Reviewer #1: The paper is good, but before publication I suggest authors to give more details about the CMOS used, including focal length of the lens, and the estimation of the speckle size produced by the imaging system. Finally, I suggest authors to consider more statistical analysis on the data to make more reliable their results.

Thank you for the comments. We have added details about the camera used and beam size and details of the speckle size. The speckle size was estimated to be 43.4μm at the observation plane or the CMOS camera. The following part has been added to the manuscript.

Fig. 2(a) shows a schematic of the laser speckle measurement system. A LD (laser diode) module of wavelength (λ) 635 nm (S2011-EC, Thorlabs, Japan) consisting of a focusing lens producing an elliptical beam size of 2.45 (a) mm × 0.54 mm was used as a light source.

The distances between the sample and the laser, as well as that between the sample and the camera, were set as 167 mm (D). Approximate speckle size (using the relation λ x D /a) was calculated to be 43.4μm (Dainty, 1975).

Considering the average speckle size at the observation plane of the CMOS camera and the pixel size of the CMOS which is around 5μm, we would like to point out that more than sufficient number of pixels covered the speckle thus ensuring enough signal-to-noise level. Actually, for a CMOS camera the readout levels determine the noise level which is 25 RMS e- per pixel for the camera used, and thus based on the pixel size and the number of pixels per speckle, the SNR of the measurements were well above the readout noise levels.

We have added statistical analysis data to add reliability to our data.

A t-test was used to investigate the significance of BA under differences for young and old leaf against control or no sound condition and the results are shown in Supplementary Table1.

Reviewer #2: Speckle imaging has been proved to be an effective way for structure measurement. In biology, it has been used to distinguish different cellular movement patterns and indicate dynamic processes. In this manuscript, authors used speckle imaging to study the interaction between plant leaves and various acoustic frequencies. They show that speckle imaging can perform real-time monitoring.

After reading this manuscript, several questions arise. I hope they can be answered appropriately.

1. Specifications of the speckle imaging system are required, including accuracy, stability, sensitivity, etc. For instance, Figure 6 claims the difference between leaves at different stages. However, it is possible that the authors merely measured the shot noise limit of the CMOS camera because all curves tend to overlap at BA=0.8. Different rising edges can be caused by different scattering ratio which may not relate to structural difference.

Thank you for the valuable comments and appreciating our application of speckles to plant-sound interaction. We have added data that support the stability and reliability of the measurement. We have added the experiment and results obtained with an inanimate paper object and showed the stability of the results. The following parts are added to the manuscript:

Furthermore, to establish the reliability of the measurement, a diffused inanimate paper sample was used to produce speckles and videos of speckles were recorded under the same sound exposure protocol used for plants to measure the biospeckle activity.

To test whether the BA variation is due to the activities of the plant, speckle videos were recorded with an inanimate paper sample under the same sound protocol of all the frequencies used. The results obtained with paper are given in Fig.5. As can be seen from the BA variation of paper, the variations obtained with paper are almost close to zero indicating no movement or indicating the stationary stare of the paper. This result also establishes that the proposed measurement technique is reliable and stable.

As for the CMOS camera used in our experiments, the noise limit is determined by the readout noise and that for our camera is 25 RMS e^-^ or in other words for a reliable readout, the CMOS pixel requires a minimum of 25 electrons per pixel. The speckle size almost covered nine pixels and therefore we believe the readout noise would not affect the speckle intensity detected. We could confirm that the CMOS was operated at relatively high levels, and we also used binning to reduce while processing the images in calculation of correlation and thus BA. Therefore, we can assure that our signals were well above the detection limit of the CMOS readout levels.

We have paid attention only to the plateau now and not exactly to the rising edge. As you point out, the rising edges could be slightly different under different frequencies. However, to carefully analyze the signal at the rising edges, we need to obtain at a higher temporal resolution. Now the frame rate is only 15fps and is not enough to look at the dynamics close to the starting edge. In future, we plan to conduct a study to look at the start a higher frame rate.

As you point out, all the BA signals plateauing at 0.8, i.e.at 20 seconds or well before at around 5 seconds. To make it clear, we have modified Figure 6 for different frequencies, with age as a parameter. As you point out, all plateau at 0.8. Therefore, we suggest whatever the effects the sound could be reaching an equilibrium as these are sum of many repetitions. The exposure to sound is for one minute followed by 20 second observation after the sound source is switched off. As you can see from comparing with an inanimate scattering object such as paper, we do not have any activity that suggests the BA signals reflect the transient activity of the plant. Of course, we can try for longer durations and continuous monitoring after each day exposure for an hour or so as done in field studies. In future, we do plan to do when the environmental conditions become permissible.

Fig.6 is revised with age as a parameter to show the age difference response to different frequencies and could be seen clearly in the revised one. Although, we still do not understand the need for this kind of differential response, by our method we could visualize such effects.

2. Phantom tests are also required to prove the performance of the system. Simple specimens, such as gel or metal, can be used to verify system performance before the biological tests. For instance, even though absorption boards are used to reduce vibration, system instability can also lead to a smaller BA when the stimuli frequency is 10 kHz. If the system can show a constant BA regardless of the stimuli frequencies when conducting phantom tests, BA values under different stimuli will be more trustworthy.

As shown in the figure above obtained with the phantom experiment of a paper under different frequencies, BA values as suggested by you are almost constant close to zero as the objects do not have any internal activity as in plants. Our phantom results did confirm the sensitivity of the biospeckle method in detection of plant-sound interactions. We appreciate your insightful comment.

3. The reason for selecting 0 Hz, 100 Hz, 1 kHz and 10 kHz needs to be stated. As shown in the Introduction, meaningful phenomena were discovered within hundreds of Hz. Instead of using high frequencies, a high frequency resolution and a range within hundreds of Hz are preferred.

We have also revised Figure 6 (currently Figure 7 shown above) to make the effect of different frequencies and the age of the plant clearer. We completely agree with the reviewer in usage of different low frequencies. As an initial experiment, experiments were done for both low, middle, and high band of frequencies. Meanwhile, because of pandemic, we had difficulty to conduct experiments themselves. As pointed out by the reviewer, we will do for lower frequencies, and, moreover, continuous long-time experiments to study the effect of sound on plant growth in future.

4. What is the necessity of creating BA? According to Eq. (2), it simply equals to 1-r, and r is the correlation coefficient.

Of course, as you say we can discuss everything in terms of the correlation coefficient. BA which is 1 -r makes the value positive so that we can discuss in terms of whether there is an increase or decrease in the internal activity of the plant based on the increase of BA. Therefore, the parameter BA was introduced out of convenience also prevalent in biospeckle research field and this is also clarified in the manuscript:

Here we would like to point out that the quantity BA was introduced so that it takes a positive value and thus we can discuss in terms of whether there is an increase or decrease in the internal activity of the plant, based on the increase of BA. Therefore, the parameter BA was introduced out of convenience, also prevalent in the biospeckle research field (Zdunek and Cybulska 2011).

5. The full name of an aberration needs to be listed when the aberration occurs first. In Line 42 and Line 95, there are missing full spells.

I apologize for some misspellings. I have corrected them.

6. Grammar errors. The Abstract itself contains many grammar errors. Several errors in the Abstract are listed in the following. Line 25, it should be “are destructive” instead of “destructive”. Line 30, it should be “intensities of speckles change over time” instead of “intensity of speckles changes in time”. Line 34, there is a missing article. Line 42, “a leaf” instead of “leaf” should be used.

Thank you for kindly pointing out the grammatical and spelling errors. Abstract was revised to be devoid of errors pointed out by the reviewer.

The Introduction and Reference sections were revised to account for several referencing discrepancies.

We have thoroughly checked, and we do hope to have the revised manuscript devoid of such errors.
