# Supplementary material for: Demonstration of laser biospeckle method for speedy in vivo evaluation of plant-sound interactions with arugula
Source: PLoS One. 2021 Oct 28;16(10):e0258973. doi: 10.1371/journal.pone.0258973 (PMC8553064; doi:10.1371/journal.pone.0258973)
Supplement: Attachment — Submitted filename: Comments.docx [file pone.0258973.s006.docx]

The authors reply my concerns properly with newly-added details and improved English writing. Compared with the former version, the latest one is more convincible. However, there are still three questions I would like the authors to expand on.

1. For the phantom test shown in Fig. 5, it looks like that the BA increases a little along with the testing time. Is it necessary to subtract the phantom results from the Arugula results to correct the final BA values? Moreover, what are the reasons for the increase in the phantom test?
2. For the LSM results shown in Fig. 10, the control group shows ratios larger than 1. As there were no sound stimuli, what are the reasons for the ratios larger than 1?
3. The gray regions in Fig. 10 are too narrow to see. The resolutions of all the figures need to be improved.
