# Supplementary material for: Demonstration of laser biospeckle method for speedy in vivo evaluation of plant-sound interactions with arugula
Source: PLoS One. 2021 Oct 28;16(10):e0258973. doi: 10.1371/journal.pone.0258973 (PMC8553064; doi:10.1371/journal.pone.0258973)
Supplement: Attachment — Submitted filename: Reply to reviewer2Comments.docx [file pone.0258973.s007.docx]

The authors reply my concerns properly with newly-added details and improved English writing. Compared with the former version, the latest one is more convincible. However, there are still three questions I would like the authors to expand on.

**Reply to comments of Reviewer 2**

1. For the phantom test shown in Fig. 5, it looks like that the BA increases a little along with the testing time. Is it necessary to subtract the phantom results from the Arugula results to correct the final BA values? Moreover, what are the reasons for the increase in the phantom test?

Thank you for the valuable comment. As you say, BA with paper seems to be increasing. We decided to take the correlation at the dark pixels of the camera over a period of 50 sec instead of 20 sec. In the graph below, we show the obtained result with an overlay of the BA obtained with paper. As you can see the red line corresponding to BA for paper is within the noise level determined by the fluctuations of the camera. This noise level is almost one tenth of BA of the signals obtained with plants and therefore could be neglected. As it is rather random, we have not

subtracted the noise level from the BA calculated for plants. The modifications has been included to the manuscript.

Although the variation shows a tendency of increase it was found to be within the noised level (Supplementary Fig.3).

Fig.S3 Results of paper shown in red overlaid with the noise from camera noise shown in dotted lines. The camera noise correlation was calculated over a total period of 50 sec.

2. For the LSM results shown in Fig. 10, the control group shows ratios larger than 1. As there were no sound stimuli, what are the reasons for the ratios larger than 1?

The ratio is calculated by dividing the stomatal sizes after by those before exposure to sound. If there is no change, it should be one. However, we found that the returning of the stomatal sizes to the values close to before exposure is not happening monotonously but more as of expansions and contractions. Therefore, the stomata tended to return to the original sizes rather more in a fluctuating manner. It is beyond the scope of this paper how the stomatal movements are controlled. We have modified the manuscript along this line as follows:

In order to make the difference cleared due to exposure, a ratio was calculated by dividing the stomatal sizes after by those before exposure to sound. If there is no change due to sound, the ratio should be close to one. However, as we can see the ratio fluctuates around one sometimes becoming larger than one. This indicates that the returning of the stomatal sizes to the values close to before exposure is not happening monotonously but more as of expansions and contractions. In other words, the stomata tended to return to the original sizes more in a fluctuating manner.

3. The gray regions in Fig. 10 are too narrow to see. The resolutions of all the figures need to be improved.

Thank you for the helpful suggestion. We have modified the figure to include the magnified change across the gray region to make the

Figure 10. The stomatal sizes, major and minor axes lengths represented respectively in red square and circle as a function of time shown on the top left and the ratio of stomatal lengths on the top right for 10kHz sound (A) and for control (B). The gray shaded regions shown at the top for the stomatal lengths and ratio are magnified to show respectively the first three seconds in the bottom row. Here the ratio is taken with the stomatal sizes measured after exposure to those before exposure.

changes clearer between the control and under sound (10kHz) conditions. The quality of the figures has also been improved.
